# Diagnostic Accuracy of Neutrophil-Creatinine Index for Predicting Severe Acute Pancreatitis Using the Revised Atlanta Classification As Gold Standard

**DOI:** 10.7759/cureus.96303

**Published:** 2025-11-07

**Authors:** Fatima Rauf, Muhammad Hanif, Huma Sabir Khan, Tashfeen Farooq, Muhammad Rawal Saeed, Romana Imtiaz, Suman Aamir, Iffat Noureen, Usman Qureshi

**Affiliations:** 1 Surgical Unit II, Benazir Bhutto Hospital, Rawalpindi Medical University, Rawalpindi, PAK; 2 Surgical Unit II, Benazir Bhutto Hospital, Rawalpindi Medical Uninversity, Rawalpindi, PAK; 3 Surgery, Regional Headquarter Hospital, Skardu, PAK; 4 Surgery Unit II, Benazir Bhutto Hospital, Rawalpindi Medical University, Rawalpindi, PAK

**Keywords:** acute pancreatitis, atlanta classification, creatinine, neutrophil-creatinine index, neutrophils, severe acute pancreatitis

## Abstract

Introduction: Early prediction of severity is important to guide treatment and triage. Many scoring systems and biomarkers are available, but none are both simple and highly accurate at admission. The aim of our study was therefore to determine the diagnostic accuracy of the neutrophil-creatinine index (NCI) in diagnosing severe acute biliary pancreatitis, using the revised Atlanta classification (2012) as the gold standard.

Methods: This cross-sectional validation study was conducted in the department of surgery, Benazir Bhutto Hospital, Rawalpindi, over a period of six months. A total of 217 patients with acute pancreatitis (AP) were included by non-probability consecutive sampling. The diagnosis was based on clinical, biochemical, and imaging criteria. The severity of AP was classified according to the revised Atlanta classification (2012), which served as the gold standard. The NCI was calculated at admission as absolute neutrophil count (× 10³/µL) × serum creatinine (mg/dL). A cut-off value of ≥11.27 was considered positive for severe AP (SAP). Diagnostic accuracy was assessed using sensitivity, specificity, predictive values, and receiver operating characteristic (ROC) curve analysis.

Results: Of 217 patients, 21 (9.7%) developed SAP. At the cut-off of 11.27, the NCI showed sensitivity 95.2%, specificity 91.3%, positive predictive value (PPV) 54.1%, negative predictive value (NPV) 99.4%, and overall accuracy 91.7%. The ROC analysis demonstrated excellent discrimination, with an area under the curve (AUC) of 0.96 (95% confidence interval (CI) 0.92-1.00).

Conclusion: The NCI is a simple and inexpensive parameter that can predict SAP with high accuracy at admission. It may serve as a practical adjunct to existing scores and biomarkers, especially in resource-limited settings.

## Introduction

Acute pancreatitis (AP) is an acute inflammatory condition of the pancreas that is characterized by abdominal pain and elevated levels of pancreatic enzymes, such as serum amylase and lipase. It may result in a wide spectrum of disease, ranging from mild and self-limiting illness to severe disease complicated by persistent organ failure and death. It is among the most common reasons for hospital admission due to gastrointestinal diseases and is associated with a significant clinical and economic burden worldwide [1].

The clinical course of AP varies widely. In most patients, the disease is mild and resolves with supportive care. However, approximately 20-30% of patients develop moderately severe AP (MSAP) or severe AP (SAP), which is associated with complications such as necrosis, sepsis, and multiorgan failure [2,3]. Mortality in mild pancreatitis is usually less than 2%, whereas SAP carries mortality rates as high as 20-30%, particularly when infected pancreatic necrosis is present [4]. The overall mortality across all forms of pancreatitis is estimated to be about 5% [5]. These statistics emphasize the importance of accurate and early risk stratification. Identifying which patients are likely to worsen is critical for guiding treatment, level of monitoring, and early interventions that may improve outcomes.

Globally, AP represents a growing health issue. Data from Europe and North America show rising incidence, partly related to gallstones, alcohol use, obesity, and hypertriglyceridemia [6]. The Global Burden of Disease (GBD) 2019 study estimated more than 2.8 million cases of AP annually worldwide, contributing significantly to morbidity and mortality [7]. Hospitalizations for AP are associated with prolonged hospital stays, high readmission rates, and high hospital costs, with AP ranking among the top gastrointestinal causes of emergency admissions [8].

Several clinical scoring systems have been developed to predict severity and guide early management. The Ranson and Glasgow scores, among the earliest tools, require 48 hours of data, delaying early prediction. Acute Physiology and Chronic Health Evaluation II (APACHE II), although comprehensive and validated in intensive care settings, requires numerous physiological and biochemical variables and repeated measurements, which limit its use in routine practice [9].

The Computed Tomography Severity Index (CTSI) incorporates imaging findings but is not used for admission triage. The Bedside Index for Severity in Acute Pancreatitis (BISAP) was developed as a simpler score based on data available within the first 24 hours. BISAP has been validated in multiple populations for predicting mortality and severity. Although useful, BISAP score has demonstrated only moderate accuracy and highlights the need for improved predictors [10].

In 2012, an international consensus group revised the Atlanta classification to standardize the definitions and severity grading of AP [11]. This classification categorizes AP into mild, moderately severe, and severe and is widely used in clinical practice. It provides uniform definitions of organ failure, local complications, and systemic complications, enabling better patient management.

Laboratory biomarkers have been extensively studied as tools for early risk stratification. C-reactive protein (CRP) is one of the most widely used inflammatory markers. While elevated CRP correlates with necrosis and poor outcomes, its levels typically peaks at 48-72 hours after symptom onset, making it less suitable for decision-making at admission [12]. Interleukin-6 (IL-6) rises earlier in the disease course and has shown good predictive accuracy for severe disease within 24 hours [13]. However, IL-6 measurement is not routinely available in all centers.

Procalcitonin (PCT) is another biomarker, particularly for predicting infected pancreatic necrosis and sepsis [14]. However, PCT assays are also relatively costly and not universally accessible.

Other hematologic indices derived from routine blood counts have also been studied. The neutrophil-to-lymphocyte ratio (NLR) and platelet-to-lymphocyte ratio (PLR) reflect systemic inflammation and immune response. Studies demonstrate that elevated NLR and PLR are associated with severe disease and mortality [15,16]. Similarly, the delta neutrophil index (DNI), which represents the fraction of immature granulocytes, has also been reported as an early predictor of severe disease [17].

Neutrophils play a central role in the pathophysiology of AP. They infiltrate pancreatic tissue early, releasing proteolytic enzymes, reactive oxygen species, and inflammatory mediators that worsen local and systemic injury. Neutrophil extracellular traps (NETs), which are networks of extracellular fibers composed of DNA and proteins, contribute to microvascular plugging and distant organ dysfunction [18]. Elevated neutrophil counts and ratios have consistently been associated with poor outcomes in AP [15,17].

Serum creatinine is another important parameter reflecting renal function and systemic involvement. Acute kidney injury is a well-known complication of SAP and is associated with high mortality. Even mild elevations in creatinine at admission have been linked to increased risk of organ failure and death [19-21].

The neutrophil-creatinine index (NCI) combines these two routine laboratory parameters by multiplying absolute neutrophil count (× 10^3^/µL) with serum creatinine (mg/dL). This simple formula is designed to represent both inflammatory activation and early renal dysfunction in a single parameter. In a study by Sahin et al., the NCI predicted SAP with sensitivity of 78.6%, specificity of 79.8%, positive predictive value (PPV) 43.7%, negative predictive value (NPV) 94.9%, and overall accuracy 79.6% at a cut-off of >11.27 [22].

On reviewing the literature, we found only one study that assessed the NCI as a predictor of severity in AP [22]. We designed this study to validate the diagnostic accuracy of the NCI. If validated, the NCI could provide clinicians with a rapid, inexpensive, and easily available marker for early identification of patients at risk of severe disease. This parameter would be particularly valuable in resource-limited settings where advanced biomarkers and imaging are not easily accessible.

The aim of our study was, therefore, to determine the diagnostic accuracy of the NCI in diagnosing severe acute biliary pancreatitis, using the revised Atlanta classification (2012) as the gold standard.

## Materials and methods

A cross-sectional validation study was conducted at the department of surgery, Benazir Bhutto Hospital, Rawalpindi, over a period of six months, from March 1, 2025, to August 31, 2025, after approval of the study synopsis and departmental review board. The hospital is a tertiary-care center that admits a large number of patients with biliary and pancreatic diseases. Standard institutional protocols, based on international guidelines for AP, are used in the management of patients [11,23]. All consecutive patients aged between 20 and 60 years who presented to the emergency or outpatient department with features of AP were screened for eligibility. Patients were included if they fulfilled at least two of the following three criteria: (1) characteristic abdominal pain of AP, defined as sudden-onset, persistent epigastric pain with or without radiation, and scored as >3 on a 10-point visual analogue scale (VAS); (2) Serum amylase and/or lipase level ≥3 times the upper limit of normal; and (3) Imaging findings consistent with AP on ultrasound or CT. Patients were excluded if they had pre-existing renal failure (serum creatinine >1.8 mg/dL), congestive cardiac failure with an ejection fraction <30%, radiological evidence of pleural effusion, intestinal perforation or bowel obstruction, or pancreatitis secondary to trauma or endoscopic retrograde cholangiopancreatography (ERCP). Patients with known chronic pancreatitis without biochemical or radiological evidence of acute inflammation were also excluded.

The sample size was calculated using sensitivity and specificity calculator, based on previously published values for the NCI. Sahin et al. reported that at a cut-off of 11.27, the NCI predicted SAP with a sensitivity of 78.6% and a specificity of 79.8% [22]. Assuming a prevalence of severe cases at 30%, a confidence level of 95%, and a margin of error of 10%, the required sample size was calculated as 217 patients [3]. A non-probability consecutive sampling technique was used. All eligible patients presenting during the study period who met the inclusion criteria were included in the study. After obtaining informed consent, demographic information (age and gender) was recorded. Blood samples were drawn at the time of admission before starting definitive treatment. Complete blood counts, including absolute neutrophil counts, were performed on automated hematology analyzers. Serum creatinine was measured using the hospital’s biochemistry analyzer. The NCI was calculated as:

$NCI = \text{Absolute neutrophil count} \; (\times 10^{3}/\mu\text{L}) \times \text{Serum creatinine} \; (\text{mg/dL})$

A cut-off of ≥11.27 was taken as SAP. Severity of AP was graded according to the revised Atlanta classification (2012): (1) Mild AP (MAP) - No organ failure and no local or systemic complications; (2) MSAP - Transient organ failure (<48 hours) and/or local complications; and (3) SAP - Persistent organ failure >48 hours, as defined by the modified Marshall scoring system [11]. This classification served as the gold standard for determining disease severity.

The primary outcome was the diagnostic accuracy of the NCI in predicting SAP compared with the revised Atlanta classification. Diagnostic accuracy was expressed in terms of sensitivity, specificity, PPV, NPV, overall accuracy and receiver operating characteristic (ROC) curve analysis with area under the curve (AUC). All data were entered into a predesigned proforma and analyzed using SPSS version 25 and Microsoft excel. Continuous variables such as age, neutrophil count, creatinine, and the NCI values were denoted as mean ± SD. Categorical variables, including gender and severity grades, were presented as frequencies and percentages. Diagnostic accuracy metrics were calculated using 2 × 2 contingency tables. Sensitivity, specificity, PPV, and NPV and overall diagnostic accuracy were calculated. In addition to descriptive statistics and diagnostic accuracy metrics, the association between the NCI values and disease severity (based on the revised Atlanta classification) was assessed using the Pearson Chi-square test. Linear-by-linear association was also calculated to evaluate the trend across increasing severity categories. A p-value of <0.05 was considered statistically significant. ROC curve was constructed in Microsoft Excel, and AUC value with 95% confidence intervals was computed. The Youden index (J; sensitivity + specificity - 1) was used to determine the optimal cut-off point. The study was approved by the departmental review board and informed consent was obtained from all participants before enrollment.

## Results

A total of 217 patients diagnosed with AP were included according to the inclusion criteria. The mean age of the study population was 41.46 ± 8.02 years. There were 72 males and 145 females, with a male-to-female ratio of approximately 1:2. Based on the revised Atlanta classification (2012), the majority of patients had MAP (n = 179), while 17 patients had moderately severe disease. 21 patients progressed to SAP. Table 1 shows the baseline demographic and clinical characteristics of patients with AP. 

**Table 1:** Baseline demographic and clinical characteristics of patients with AP NCI: Neutrophil-creatinine index; MAP: Mild acute pancreatitis; MSAP: Moderately severe acute pancreatitis; SAP: Severe acute pancreatitis; AP: Acute pancreatitis

Variable	Value
Total patients (n)	217
Mean age (years)	41.46 ± 8.02
Mean neutrophil count (× 10³/µL)	7.22 ± 2.90
Mean creatinine (mg/dL)	0.79 ± 0.54
Mean NCI	6.21 ± 5.09
Sex distribution	
Male	72 (33.2%)
Female	145 (66.8%)
Severity of AP (Atlanta classification (2012))	
MAP	179 (82.5%)
MSAP	17 (7.8%)
SAP	21 (9.7%)

The diagnostic accuracy of the NCI was assessed using a cut-off value of ≥11.27. The revised Atlanta classification was taken as the gold standard for defining severity of disease. Table 2 demonstrates the 2 × 2 contingency table used to calculate the sensitivity, specificity, PPV, and NPV of the NCI cut-off against the revised Atlanta classification.

**Table 2:** 2 × 2 contingency table NCI: Neutrophil-creatinine index; SAP: Severe acute pancreatitis; MSAP: Moderately severe acute pancreatitis; MAP: Moderate acute pancreatitis

Atlanta classification (gold standard)	NCI severe (≥11.27)	NCI non-severe (<11.27)	Total
Severe (SAP)	20 (True Positive)	1 (False Negative)	21
Non-severe (MAP/MSAP)	17 (False Positive)	179 (True Negative)	196
Total	37	180	217
Pearson Chi-square	χ² = 400.375, df = 292, p < 0.001		
Linear-by-linear association	χ² = 114.135, p < 0.001		

Sensitivity was 95.2%, specificity was 91.3%, PPV was 54.1%, NPV was 99.4%, and overall accuracy was 91.7%. J was 0.87, further confirming the strong balance between sensitivity and specificity of the NCI cut-off (≥11.27).

Chi-square analysis demonstrated a statistically significant association between the NCI and the revised Atlanta classification. The Pearson Chi-square test was highly significant (χ² = 400.375, df = 292, p < 0.001), and the linear-by-linear association was also significant (χ² = 114.135, p < 0.001). These results confirm that the NCI is not only an accurate diagnostic marker, but also that its values are strongly correlated with disease severity categories, supporting its role as a reliable parameter for stratifying patients with AP.

**Figure 1:**
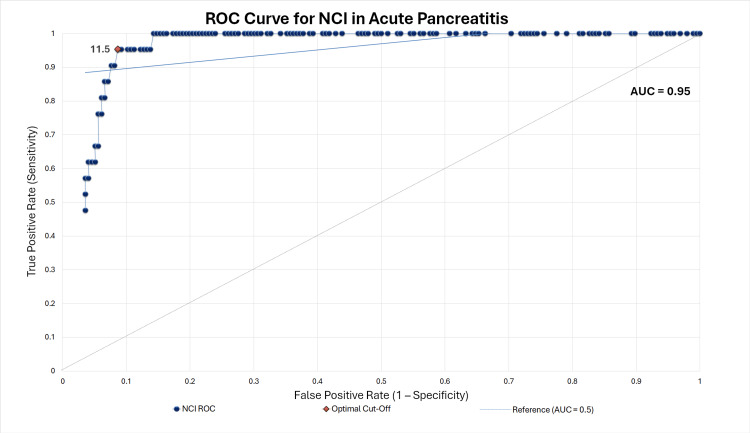
ROC curve of the NCI for the prediction of SAP ROC: Receiver operating characteristic; NCI: Neutrophil-creatinine index; SAP: Severe acute pancreatitis

ROC curve analysis confirmed excellent discriminatory ability. The ROC curve was constructed in Microsoft Excel, and the AUC value with 95% confidence interval (CI) was computed. J (sensitivity + specificity - 1) was applied to determine the optimal cut-off point. The previously proposed cut-off value of 11.27 was validated in this study, while an additional optimal cut-off of 11.5 was also identified, which provided the same sensitivity (95.2%) and specificity (91.3%). The overall AUC was 0.96 (95% CI 0.92-1.00), reflecting very high diagnostic performance (Figure 1). The red marker in Figure 1 highlights the optimal cut-off point on the ROC curve.

## Discussion

In our study, we evaluated the diagnostic accuracy of the NCI for predicting SAP using the revised Atlanta classification (2012) as the gold standard. We found that the NCI at admission demonstrated high sensitivity (95.2%), high specificity (91.3%), and excellent discrimination with an AUC of 0.96 at a cut-off of ≥11.27. Notably, the NPV was 99.4%, indicating that patients with the NCI values below this threshold were highly unlikely to develop severe disease. These findings suggest that the NCI is a reliable and inexpensive parameter for excluding severe disease at admission. In clinical practice, this parameter could help guide decisions about management of patients requiring intensive care and monitoring, particularly in resource-limited settings.

Our results compare favorably with existing scoring systems used in the early assessment of AP. Ranson’s criteria and the Glasgow score, while important, require 48 hours to complete and are therefore less useful for making a decision at the time of admission. The APACHE II score, though validated, is cumbersome to apply and requires multiple physiologic and biochemical parameters [9]. BISAP is a simpler tool that can be calculated within 24 hours but has moderate accuracy [10]. The CTSI provides additional information on local complications but is not intended for early use and is less feasible in unstable patients [12].

Inflammatory biomarkers are widely studied for their prognostic utility in AP. CRP is the most established marker, but it peaks after 48-72 hours and thus has limited utility as an admission tool [13]. IL-6 rises earlier and has shown good predictive accuracy for severe disease [14]. However, IL-6 measurement is not routinely available. PCT is particularly useful in predicting infected pancreatic necrosis, with pooled AUCs around 0.80-0.86 [6]. Like IL-6, its routine use is limited by cost and availability. Our study demonstrates that the NCI compares favorably with these biomarkers, offering equal or even better accuracy than IL-6, CRP, and PCT. More importantly, the NCI is calculated using absolute neutrophil count and serum creatinine that are universally available and inexpensive. This makes the NCI a useful option in both resource-rich and resource-limited healthcare settings.

The high accuracy of the NCI is supported by disease pathophysiology. Neutrophils respond early in AP. They infiltrate the pancreas, release proteolytic enzymes and reactive oxygen species, and form NETs, which contribute to systemic inflammation and multiorgan failure [18]. Elevated neutrophil counts and neutrophil-derived indices such as the neutrophil-to-lymphocyte ratio (NLR) and DNI have been associated with poor outcomes in AP [17,24]. Elevated serum creatinine shows systemic involvement. Even mild increases in creatinine at admission are associated with higher risk of persistent organ failure and mortality [20,21]. By combining these two parameters, the NCI denotes both early inflammatory activity and early evidence of organ dysfunction. This dual representation likely explains its superior predictive accuracy compared with other single markers.

In addition to the diagnostic accuracy of the NCI at the previously proposed threshold of 11.27, our analysis also identified an additional optimal cut-off of 11.5 using J. This value yielded identical sensitivity and specificity, highlighting that the predictive performance of the NCI is uniform across a narrow threshold range and unlikely to be affected by minor laboratory or population differences. Importantly, Chi-square testing demonstrated a highly significant association (p < 0.001) between the NCI and the revised Atlanta classification. These findings not only validate the NCI as a diagnostic tool but also confirm that it is in line with the Atlanta classification which is currently the gold-standard in determining the severity of AP, thus supporting its integration into routine clinical practice.

The findings of our study have many clinical implications. Firstly, the NCI can be easily calculated at the time of admission, making it practical for early triage. Moreover, patients with high NCI values can be managed under closer observation and active supportive measures such as aggressive fluid resuscitation and nutritional support. However, it should be used in conjunction with other markers in determining the course of disease and guiding further management.

The strengths of this study include its practical design, use of the revised Atlanta classification as the gold standard, and clear diagnostic accuracy metrics based on contingency analysis. However, our study was limited by its single-center design which limits generalizability. The proportion of severe cases in our study (9.7%) was slightly lower than some international series, which may have influenced predictive values. Moreover, we did not directly compare the NCI with other parameters like BISAP or APACHE II scores within the same dataset. Advanced biomarkers such as IL-6 and PCT were not measured in all patients due to limited resources which also affected the direct comparison among the markers. Finally, the NCI was only assessed at admission, while serial measurements may provide additional prognostic value.

Future research should aim to validate the NCI in larger, multicenter cohorts. Direct comparisons with BISAP, APACHE II, and other scoring systems would clarify its relative performance. Studies incorporating IL-6, CRP, and PCT alongside the NCI would allow development of integrated, multimarker prediction models. Cost-effectiveness analyses should also be performed, particularly in low- and middle-income countries where advanced biomarkers are not available and ICU resources are limited.

## Conclusions

This study showed that the NCI, calculated at admission, is a highly accurate parameter for predicting SAP. With a sensitivity of 95.2%, specificity of 91.3%, and an AUC of 0.96, the NCI demonstrated excellent diagnostic performance.

Because the NCI is based on two routine and inexpensive laboratory tests, it is practical for use in both advanced and resource-limited healthcare settings. Its simplicity makes it a useful addition to current severity assessment methods. Wider validation in larger, multicenter studies is needed, along with comparisons to BISAP, APACHE II, and other biomarkers. If confirmed, the NCI could become part of everyday clinical practice to improve the management of patients, reduce complications, and ultimately lower the mortality in patients of AP.
